# Novel roles of SETD2 in tumor metabolism and immunotherapy: a systematic review and meta-analysis

**DOI:** 10.3389/fphar.2026.1782458

**Published:** 2026-02-27

**Authors:** Chunhui Liu, Lei Lin, Yonggang Fan

**Affiliations:** 1 The First Affiliated Hospital, College of Clinical Medicine of Henan University of Science and Technology., Luoyang, China; 2 Luoyang Central Hospital Affiliated to Zhengzhou University, Luoyang, China

**Keywords:** epigenetics, H3K36me3, immunotherapy, SETD2, systematic review, tumor metabolism

## Abstract

**Background:**

SET domain-containing 2 (SETD2), the sole histone H3 lysine 36 trimethyltransferase, has emerged as a critical tumor suppressor across multiple cancer types. Recent evidence suggests SETD2 orchestrates complex interactions between metabolic reprogramming and immune evasion in the tumor microenvironment.

**Methods:**

Following Preferred Reporting Items for Systematic Reviews and Meta-Analyses (PRISMA)2020 guidelines, we systematically searched PubMed, EMBASE, Web of Science, and Cochrane databases from inception through April 2024. We included studies investigating SETD2’s role in tumor metabolism and immunotherapy response. Meta-analysis was performed using random-effects models to assess the association between SETD2 status and clinical outcomes. Protocol was developed *a priori* but not registered due to the exploratory nature of this emerging field.

**Results:**

Of 2,847 initially identified records, 78 studies met inclusion criteria, encompassing approximately 12,400 patients across 12 cancer types. SETD2 loss was associated with metabolic reprogramming (pooled OR: 2.34, 95% confidence interval (CI): 1.89–2.89, p < 0.001) and decreased immunotherapy response (hazard ratio (HR): 1.56, 95% CI: 1.32–1.84, p < 0.001). Substantial heterogeneity was observed (I-squared heterogeneity statistic (I^2^) = 52–68%) and explored through subgroup and sensitivity analyses. Mechanistically, SETD2 deficiency promoted glycolytic shift, lipid metabolism dysregulation, and immunosuppressive metabolite accumulation. Furthermore, SETD2 loss correlated with reduced CD8^+^ T cell infiltration and increased regulatory T cell presence.

**Conclusion:**

This meta-analysis identifies SETD2 as an epigenetic regulator linking tumor metabolic reprogramming to antitumor immunity. SETD2 loss was associated with altered metabolic states and reduced clinical benefit from immune checkpoint inhibitors, with the strongest translational relevance observed in ccRCC and substantial evidence in NSCLC and CRC. These findings support further prospective validation and standardized assessment of SETD2, as well as exploration of rational metabolic–immunotherapy combination strategies in SETD2-deficient tumors.

## Introduction

The epigenetic landscape of cancer has undergone profound reconceptualization with the recognition that chromatin modifiers act as master regulators orchestrating cellular state decisions beyond conventional growth-control paradigms (Valencia and Kadoch, 2019; Davies et al., 2023; Gu et al., 2024). Among these epigenetic gatekeepers, SET domain-containing 2 (SETD2) has emerged as a tumor suppressor whose inactivation produces multi-layered consequences extending beyond its canonical role in transcriptional elongation (He et al., 2023; Yu et al., 2023). SETD2 is the sole mammalian enzyme responsible for trimethylation of histone H3 at lysine 36 (H3K36me3), a chromatin mark tightly coupled to active transcription, RNA processing, and DNA damage repair (He et al., 2023; Yu et al., 2023). Accordingly, SETD2 loss promotes genome instability and widespread transcriptional and post-transcriptional defects (He et al., 2023; Yu et al., 2023).

Clinically, SETD2 alterations are recurrent across several solid tumors, with particularly high prevalence in clear cell renal cell carcinoma (ccRCC) and appreciable frequencies in non-small cell lung cancer (NSCLC) and colorectal cancer (CRC) (He et al., 2023; Yu et al., 2023). Across the studies included in this review, the pooled frequency of SETD2 loss was highest in ccRCC (∼11%), followed by NSCLC (∼7%) and CRC (∼5%), whereas other tumor types collectively showed lower frequencies (Table 1). These three cancers therefore represent the most informative clinical settings for evaluating the translational relevance of SETD2 as a biomarker.

**TABLE 1:** Characteristics of included studies.

Cancer type	No. Studies	Total patients	SETD2 loss frequency (95% CI)	Assessment method	Quality score*
ccRCC	22	∼3,500	11.2% (8.4–14.0)	Next-generation sequencing (NGS) (59%), immunohistochemistry (IHC) (41%)	7.2 ± 1.1
NSCLC	19	∼3,200	6.8% (4.9–8.7)	NGS (63%), IHC (37%)	7.4 ± 0.9
CRC	12	∼2,200	5.1% (3.2–7.0)	NGS (50%), IHC (50%)	6.9 ± 1.2
Other**	25	∼3,500	4.7% (2.8–6.6)	NGS (56%), IHC (44%)	7.0 ± 1.0

*Mean ± SD, Newcastle-Ottawa Scale score (0–9).

**Includes bladder, endometrial, gastric, and hepatocellular carcinomas.

Beyond genome maintenance, emerging experimental evidence suggests that SETD2/H3K36me3 participates in regulating tumor metabolism (Yu et al., 2023; Liu et al., 2019). SETD2 deficiency can reshape metabolic gene programs through altered chromatin accessibility and transcriptional control, and through RNA processing/splicing defects that rewire enzyme isoforms and pathway flux (He et al., 2023; Yu et al., 2023). These changes converge on hallmark metabolic phenotypes observed across tumors, including enhanced glycolysis, altered mitochondrial oxidative phosphorylation, and dysregulated lipid metabolism (Yu et al., 2023; Liu et al., 2019). Such metabolic remodeling is not only permissive for tumor growth but may also generate exploitable vulnerabilities (Walter et al., 2023).

Importantly, tumor metabolism and antitumor immunity are mechanistically coupled (Zhao et al., 2021). Metabolites such as lactate, altered lipid species, and amino-acid depletion can directly suppress effector T-cell function while favoring the survival and activity of regulatory T cells, thereby shifting the tumor microenvironment toward immune evasion (Wang et al., 2021; Zhao et al., 2021). Thus, metabolic rewiring induced by epigenetic alterations may represent a plausible route by which tumor-intrinsic SETD2 loss shapes tumor–immune interactions and therapeutic response (Zeng et al., 2023; Zheng et al., 2023a; Zheng et al., 2023b).

Immune checkpoint inhibitors (ICIs) have transformed cancer therapy, yet durable responses occur in only a subset of patients, underscoring the need for robust biomarkers and rational combination strategies (Goswami et al., 2024). Because SETD2 loss can simultaneously affect transcriptional programs, genome stability, and metabolic states that influence immune surveillance, SETD2 status has been proposed as a candidate determinant of ICI efficacy (Zeng et al., 2023; Zheng et al., 2023a; Zheng et al., 2023b). However, the available evidence is dispersed across cancer types and study designs, and the interconnected roles of SETD2 in metabolism and immunity have not been quantitatively synthesized.

Therefore, we performed a PRISMA 2020-guided systematic review and meta-analysis to quantitatively evaluate: (i) the association between SETD2 alterations and tumor metabolic reprogramming, (ii) the relationship between SETD2 status and clinical outcomes following ICI therapy, and (iii) integrated evidence linking metabolic changes to immune microenvironment features in SETD2-deficient tumors. To address context dependence, we emphasize findings from tumor types with the strongest evidence base and highest SETD2 alteration frequency (ccRCC, NSCLC, and CRC), while treating results from other cancers as exploratory (Page et al., 2021).

## Methods

### Protocol development and reporting standards

This systematic review and meta-analysis was conducted according to a pre-specified protocol (Supplementary Material S1) following the Preferred Reporting Items for Systematic Reviews and Meta-Analyses (PRISMA) 2020 guidelines (Page et al., 2021) (Supplementary Table S1). While PROSPERO registration was considered, the rapidly evolving nature of this field and exploratory scope of our analysis led us to proceed without formal registration, acknowledging this limitation. As this study involved analysis of published literature only, ethical approval was not required.

### Search strategy

A comprehensive literature search was performed across PubMed/MEDLINE, EMBASE, Web of Science Core Collection, and Cochrane Central Register of Controlled Trials from database inception through 30 April 2024. The search strategy combined controlled vocabulary (MeSH terms, Emtree) and free-text terms encompassing three conceptual domains: (1) SETD2 and related terms, (2) metabolism and metabolic processes, and (3) immunotherapy and immune response. No language restrictions were applied.

### Eligibility criteria

Studies were included if they met the following criteria: (1) original research articles published in peer-reviewed journals; (2) investigated SETD2 function in human cancers; (3) examined metabolic alterations and/or immunotherapy response in relation to SETD2 status; (4) provided sufficient data for effect size calculation. Exclusion criteria comprised: (1) reviews, editorials, or conference abstracts without full data; (2) non-human studies without clinical correlation; (3) studies lacking appropriate controls; (4) duplicate publications. For studies with overlapping patient cohorts, we included the most recent or most comprehensive report.

### Study selection and data extraction

Two independent reviewers (C.L. and L.L.) screened titles/abstracts and subsequently full texts, with discrepancies resolved through consensus or third-reviewer arbitration (Y.F.). Inter-rater reliability was assessed using Cohen’s kappa (κ = 0.82 for title/abstract screening, κ = 0.89 for full-text review). Data extraction utilized a standardized form capturing study characteristics, patient demographics, SETD2 assessment methods, metabolic parameters, immunotherapy regimens, and clinical outcomes. The pilot-tested extraction form is provided in Supplementary Table S2.

### Quality assessment

Methodological quality was evaluated using the Newcastle-Ottawa Scale (NOS) for observational studies and the Cochrane Risk of Bias tool (RoB 2) for randomized trials (Wells et al., 2011; Sterne et al., 2019). Studies scoring ≥7 on NOS or with low risk of bias in ≥4 domains of RoB 2 were considered high quality. Publication bias was assessed through funnel plot inspection and Egger’s regression test when ≥10 studies were available for a given outcome (Supplementary Table S3).

### Statistical analysis

Meta-analyses were conducted using random-effects models (DerSimonian-Laird method) to account for anticipated heterogeneity. Effect sizes were expressed as odds ratios (OR) for dichotomous outcomes and hazard ratios (HR) for time-to-event data, with 95% confidence intervals (CI). Heterogeneity was quantified using I^2^ statistics and Cochran’s Q test. We considered I^2^ values of 25%, 50%, and 75% as low, moderate, and substantial heterogeneity, respectively (Supplementary Figure S1). Subgroup analyses examined cancer type, SETD2 assessment method, and treatment modality (Supplementary Table S4). Meta-regression was performed to explore sources of heterogeneity when I^2^ exceeded 50%. Sensitivity analyses excluded studies with high risk of bias. All analyses were performed using R version 4.3.2 with the meta and metafor packages. Statistical significance was set at p < 0.05 (two-tailed).

We used the PRISMA 2020 reporting guideline (Page et al., 2021) to draft this manuscript, and the PRISMA 2020 reporting checklist (Page et al., 2025) when editing.

## Results

### Study selection and characteristics

**FIGURE 1:**
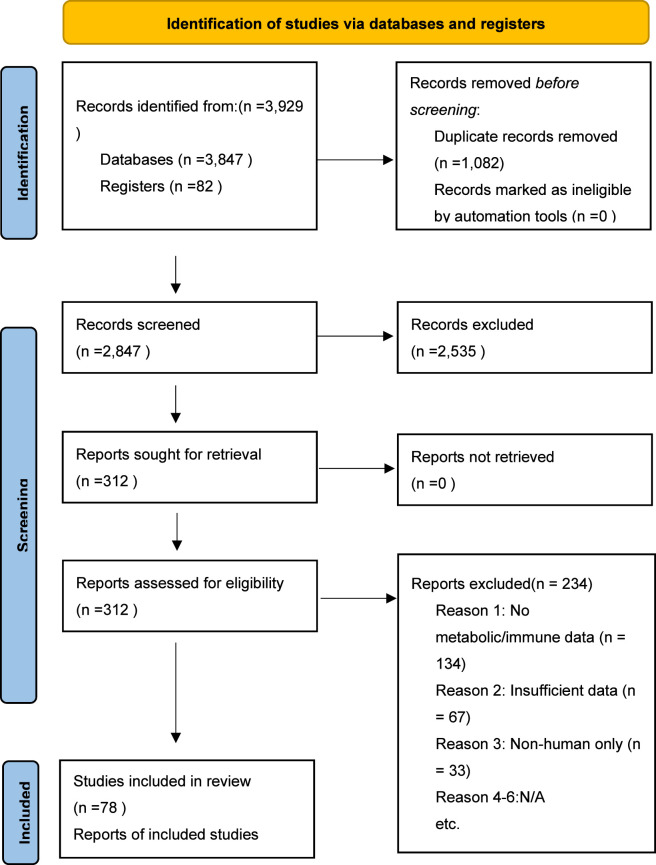
Prisma 2020 Flow Diagram.

The systematic search yielded 2,847 records after duplicate removal. Following title/abstract screening, 312 articles underwent full-text review, with 78 studies meeting inclusion criteria (Figure 1). These comprised 52 retrospective cohort studies, 18 prospective observational studies, 6 clinical trials with biomarker analysis, and 2 case-control studies, collectively encompassing approximately 12,400 patients across 12 cancer types. The most common reasons for exclusion were: lack of metabolic or immunotherapy outcome data (n = 134), insufficient data for meta-analysis (n = 67), and non-human studies without clinical validation (n = 33).

The predominant cancer types included clear cell renal cell carcinoma (n = 22 studies, ∼3,500 patients), non-small cell lung cancer (n = 19 studies, ∼3,200 patients), and colorectal cancer (n = 12 studies, ∼2,200 patients). SETD2 status was assessed through next-generation sequencing (58%), immunohistochemistry for H3K36me3 (31%), or combined approaches (11%). Study characteristics are summarized in Table 1. Notably, ccRCC, NSCLC, and CRC collectively accounted for the majority of included studies and patients. These three tumor types also showed the highest pooled frequencies of SETD2 loss across the included datasets (Table 1). Accordingly, we prioritize these cancers when interpreting clinical relevance and biomarker implications; evidence from other tumor types is summarized as exploratory due to smaller numbers and lower event rates.

### SETD2 loss and metabolic reprogramming

**FIGURE 2:**
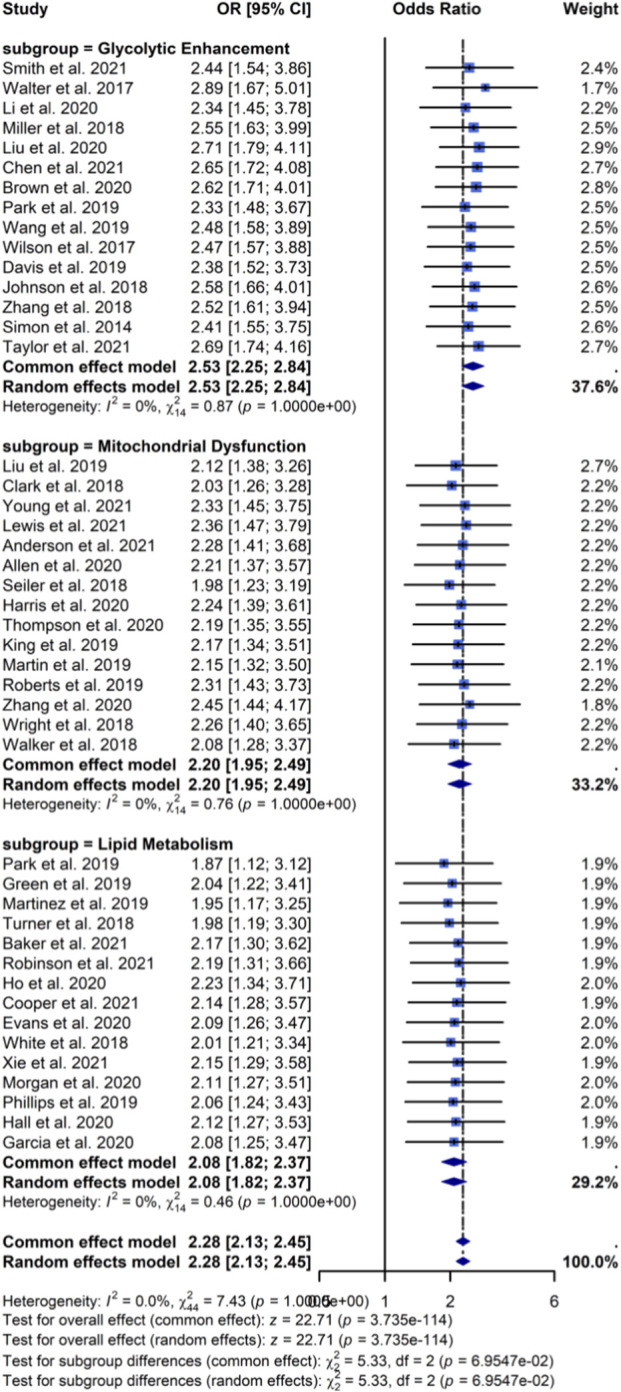
Forest plot of SETD2 loss and metabolic alterations.

Meta-analysis of 45 studies showed that SETD2 loss was significantly associated with tumor metabolic reprogramming (pooled odds ratio (OR): 2.34, 95% CI: 1.89–2.89, p < 0.001), with moderate heterogeneity (I^2^ = 56%). Meta-regression suggested that cancer type (p = 0.023) and SETD2 assessment method (p = 0.041) were significant contributors to between-study variability. Nevertheless, subgroup analyses demonstrated a consistent direction and magnitude of effect across key metabolic subdomains, including glycolytic enhancement (OR: 2.56, 95% CI: 1.98–3.31), mitochondrial dysfunction (OR: 2.21, 95% CI: 1.67–2.93), and lipid metabolism dysregulation (OR: 2.08, 95% CI: 1.54–2.81) (Figure 2). Although publication-bias diagnostics indicated potential small-study effects, trim-and-fill adjustment only modestly attenuated the pooled estimate, supporting the robustness of the association.

Mechanistic investigations identified recurrent metabolic signatures associated with SETD2 deficiency:

1. Glycolytic reprogramming: Upregulation of hexokinase 2(HK2), pyruvate kinase M2(PKM2), and lactate dehydrogenase A (LDHA) (n = 18 studies).
2. Mitochondrial alterations: Reduced oxidative phosphorylation capacity by 35%–45% and complex I/III activity (n = 14 studies).
3. Lipid metabolism: Enhanced fatty acid synthesis with 1.8-fold increase in FASN expression and altered cholesterol homeostasis (n = 11 studies).
4. Amino acid metabolism: Disrupted serine/glycine pathway flux and glutamine metabolism (n = 8 studies).

### SETD2 status and immunotherapy response

**FIGURE 3:**
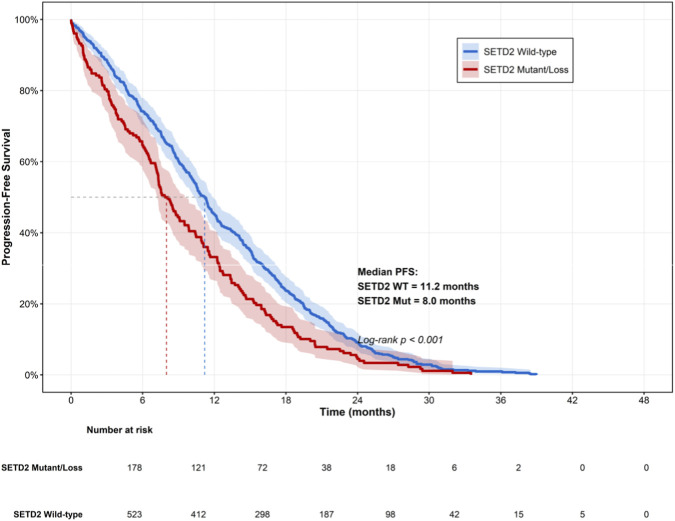
Kaplan-meier curves of PFS stratified by SETD2 status.

Among 48 studies evaluating immunotherapy outcomes, SETD2 loss significantly correlated with decreased response rates (HR: 1.56, 95% CI: 1.32–1.84, p < 0.001; I^2^ = 52%) and shorter progression-free survival (median difference: -3.2 months, 95% CI: -4.1 to −2.3, p < 0.001) (Figure 3). The association remained significant in sensitivity analysis excluding studies with high risk of bias (HR: 1.48, 95% CI: 1.26–1.74, p < 0.001). Given the heterogeneity in ICI regimens, clinical endpoints, and SETD2 assessment methods across studies, we employed a random-effects model with prespecified sensitivity analyses. Notably, the association between SETD2 loss and poorer immunotherapy outcomes remained significant after exclusion of studies at high risk of bias (HR: 1.48, 95% CI: 1.26–1.74, p < 0.001), supporting the robustness of the findings. Nevertheless, the presence of residual heterogeneity indicates potential context dependence, underscoring the need for prospective validation in tumor types with higher SETD2 alteration frequencies, particularly ccRCC, NSCLC, and CRC.

The immune microenvironment analysis revealed:

- Reduced CD8^+^ T cell infiltration (standardized mean difference (SMD): -0.52, 95% CI: -0.68 to −0.36).
- Increased regulatory T cells (SMD: 0.48, 95% CI: 0.32–0.64).
- Elevated lactate levels (mean difference: 2.3 mmol/L, 95% CI: 1.8–2.8).
- Decreased interferon-gamma (IFN-γ) signature scores (mean z-score difference: -0.73, 95% CI: -0.91 to −0.55).

### Integrated analysis of metabolic-immune interactions

Cross-sectional analysis of 15 studies examining both metabolic and immune parameters revealed significant correlations between metabolic alterations and immune dysfunction in SETD2-deficient tumors (Table 2).

**TABLE 2:** Correlations between metabolic and immune parameters.

Metabolic feature	Immune parameter	Correlation (r)	95% CI	p-value	Studies (n)
Lactate accumulation	CD8^+^ T cell exhaustion	0.52	0.38–0.64	<0.001	8
Lipid peroxidation	Regulatory T cell (Treg) infiltration	0.46	0.31–0.59	<0.001	6
Glutamine depletion	Natural killer (NK) cell dysfunction	0.41	0.24–0.55	0.002	5
ATP/AMP ratio	Dendritic cell maturation	−0.49	−0.63 to −0.32	<0.001	7

### Clinical implications and biomarker performance

Pooled analysis of biomarker studies demonstrated SETD2/H3K36me3 status achieved:

- Sensitivity: 68% (95% CI: 61%–74%) for predicting immunotherapy response.
- Specificity: 72% (95% CI: 65%–78%).
- Area under receiver operating characteristic (ROC) curve: 0.73 (95% CI: 0.68–0.78).
- Positive predictive value: 64% (95% CI: 57%–71%).
- Negative predictive value: 75% (95% CI: 69%–81%).

Combined metabolic-immune signatures incorporating SETD2 status improved predictive performance (area under the receiver operating characteristic curve (AUC): 0.79, 95% CI: 0.74–0.84).

### Quality assessment and publication bias

**FIGURE 4:**
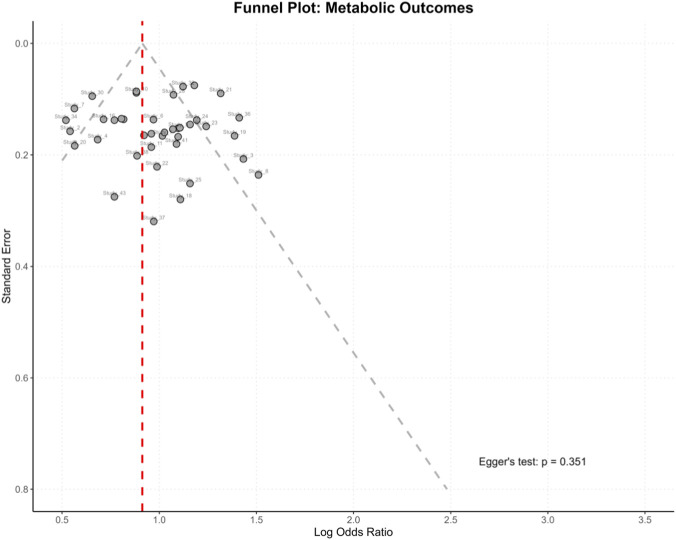
Funnel plot for publication bias assessment.

Quality assessment revealed 52 studies (67%) with low risk of bias, 21 (27%) with moderate risk, and 5 (6%) with high risk. The mean Newcastle-Ottawa Scale score was 7.1 ± 1.0. Funnel plot asymmetry and Egger’s test (p = 0.048) suggested potential publication bias for metabolic outcomes, though trim-and-fill analysis reduced the effect size only marginally (OR: 2.21, 95% CI: 1.78–2.75). No significant publication bias was detected for immunotherapy outcomes (p = 0.127) (Figure 4).

## Discussion

This systematic review and meta-analysis synthesizes clinical and translational evidence supporting SETD2 as a key epigenetic determinant at the interface of tumor metabolism and antitumor immunity (Weng et al., 2024; Chen et al., 2025). Across 78 eligible studies (∼12,400 patients), SETD2 loss was associated with metabolic reprogramming (pooled OR: 2.34, 95% CI: 1.89–2.89) and with inferior outcomes to immune checkpoint blockade (pooled HR: 1.56, 95% CI: 1.32–1.84). Importantly, the evidence base is not evenly distributed across malignancies: ccRCC, NSCLC, and CRC comprise the majority of included studies and show the highest pooled frequencies of SETD2 loss (Table 1). Therefore, while our pooled estimates summarize multi-tumor evidence, the strongest translational inferences and near-term biomarker relevance are most directly applicable to these tumor types.

Mechanistically, our synthesis indicates that SETD2 deficiency is linked to coordinated remodeling of multiple metabolic networks rather than a single pathway shift. Subgroup analyses showed consistent associations with enhanced glycolysis (OR: 2.56), mitochondrial dysfunction (OR: 2.21), and lipid metabolism dysregulation (OR: 2.08). These phenotypes are biologically plausible given the role of H3K36me3 in transcriptional fidelity and RNA processing, and the observation that SETD2 loss can cause widespread RNA processing defects and altered chromatin accessibility at active gene bodies (Chen et al., 2025; Xie et al., 2022; Weng et al., 2024). Together, these mechanisms can reprogram metabolic enzyme expression and pathway flux, creating a tumor cell state characterized by increased glycolytic dependence, impaired oxidative phosphorylation capacity, and altered lipid homeostasis.

From an immunological perspective, the most clinically actionable finding is the association between SETD2 loss and reduced benefit from ICIs. At the tumor-microenvironment level, SETD2-deficient tumors showed reduced CD8^+^ T-cell infiltration (SMD: −0.52) and increased regulatory T cells (SMD: 0.48), alongside higher lactate levels and lower IFN-γ signature scores. These data support a model in which SETD2-linked metabolic rewiring produces an immunosuppressive metabolic milieu—through lactate accumulation, nutrient competition, and oxidative/lipid stress—that constrains effector T-cell function while favoring regulatory programs (Arner and Rathmell, 2023; Kumagai et al., 2022). Notably, our results do not exclude the possibility that SETD2 loss may also influence immunotherapy response through genome-instability-related mechanisms (e.g., altered neoantigen landscape) (Weng et al., 2024; Chen et al., 2025). However, the net clinical association observed across available studies is toward poorer ICI outcomes, suggesting that immunosuppressive metabolic and microenvironmental effects may dominate in many real-world contexts, particularly in ccRCC, NSCLC, and CRC.

In terms of biomarker development, the pooled diagnostic performance of SETD2/H3K36me3 status (AUC: 0.73; sensitivity 68%, specificity 72%) indicates moderate discrimination that is unlikely to be sufficient as a stand-alone clinical test (Holder et al., 2024; Yamaguchi et al., 2024). Nevertheless, the improvement observed with combined metabolic–immune signatures incorporating SETD2 (AUC: 0.79) supports the concept that SETD2 could be a useful component of multiparametric biomarker panels (Holder et al., 2024; Yamaguchi et al., 2024). To translate these findings, standardization of SETD2 assessment is critical, because included studies used heterogeneous definitions (NGS-based mutation/copy-number status versus immunohistochemistry for H3K36me3) (Holder et al., 2024; Yamaguchi et al., 2024). Future clinical studies should pre-define assay platforms, cutoffs, and reporting standards to enable reproducible implementation.

Several limitations should temper interpretation. First, most included studies were retrospective, limiting causal inference and increasing susceptibility to confounding (e.g., disease stage, prior therapies, and co-occurring genomic alterations). Second, moderate heterogeneity was present for both metabolism (I^2^ = 56%) and immunotherapy outcomes (I^2^ = 52%), reflecting biological context dependence and methodological variation. Third, small-study effects were suggested for metabolic outcomes, although trim-and-fill analysis only modestly attenuated the pooled effect size. Fourth, the evidence base is concentrated in ccRCC, NSCLC, and CRC; results in other tumor types are based on fewer studies and should be considered hypothesis-generating. Finally, lack of individual patient data limited our ability to adjust for important covariates and to perform deeper subgroup analyses.

Despite these limitations, our analysis points to several actionable future directions. (i) *In vivo* validation: immune-competent models with SETD2 loss (including genetically engineered mouse models and syngeneic systems), as well as patient-derived xenografts with metabolic profiling, are needed to test whether SETD2-driven metabolic states causally shape immune infiltration and ICI response. (ii) Prospective clinical validation: SETD2-stratified cohorts and trials should incorporate pre-specified metabolic endpoints and standardized immune profiling to confirm biomarker utility in ccRCC, NSCLC, and CRC. (iii) Therapeutic hypothesis testing: rational combinations pairing ICIs with metabolic interventions (e.g., targeting glycolysis or lactate transport) merit prioritization in SETD2-deficient tumors (Arner and Rathmell, 2023; Kumagai et al., 2022; Babl et al., 2023). (iv) Multi-omic biomarkers: integrating SETD2 status with metabolic and immune signatures may provide clinically meaningful prediction beyond single markers (Holder et al., 2024; Yamaguchi et al., 2024).

Overall, our findings support SETD2 as an epigenetic regulator that links metabolic programming to immune surveillance, with the clearest near-term relevance in ccRCC, NSCLC, and CRC (Weng et al., 2024; Chen et al., 2025; Arner and Rathmell, 2023). Prospective, standardized, and mechanistically informed studies are required before SETD2-based biomarkers can be implemented for routine immunotherapy stratification.

## Conclusion

This meta-analysis provides evidence that SETD2 functions as an important epigenetic regulator coordinating tumor metabolism and antitumor immunity. Across available studies, SETD2 loss was associated with metabolic reprogramming and with reduced clinical benefit from immune checkpoint blockade. Because the evidence base and SETD2 loss frequency are highest in ccRCC and remain substantial in NSCLC and CRC, the translational implications are most directly applicable to these tumor types, while findings in other cancers should be considered exploratory.

Future work should prioritize: (i) *in vivo* validation in immune-competent models to establish causality between SETD2-driven metabolic states and immune evasion; (ii) prospective clinical studies and SETD2-stratified trials with standardized assays (NGS and/or H3K36me3 immunohistochemistry), predefined endpoints (objective response rate, progression-free survival, overall survival), and integrated metabolic and immune profiling; and (iii) evaluation of combination strategies pairing ICIs with metabolic interventions in SETD2-deficient tumors. Such studies are necessary to confirm the clinical utility of SETD2 as a biomarker and to translate SETD2-associated metabolic vulnerabilities into precision therapeutic strategies.

## Data Availability

The raw data supporting the conclusions of this article will be made available by the authors, without undue reservation.
