# Supplementary material for: Novel roles of SETD2 in tumor metabolism and immunotherapy: a systematic review and meta-analysis
Source: Front Pharmacol. 2026 Feb 27;17:1782458. doi: 10.3389/fphar.2026.1782458 (PMC12983094; doi:10.3389/fphar.2026.1782458)
Supplement: Supplementary file 1 [file DataSheet1.doc]

## Supplementary Materials

### Supplementary Material S1: Study Protocol (Available Upon Request)

The complete protocol for this systematic review and meta-analysis is available from the corresponding author upon reasonable request. The protocol includes detailed methodology, search strategies, data extraction forms, and statistical analysis plans developed a priori.

### Supplementary Table S1: PRISMA_2020_checklist

| **Section and Topic** | **Item #** | **Checklist item** | **Location where item is reported** |
| --- | --- | --- | --- |
| **TITLE** | | |  |
| Title | 1 | Identify the report as a systematic review and meta-analysis. | Title page (title includes “A Systematic Review and Meta-Analysis”) |
| **ABSTRACT** | | |  |
| Abstract | 2 | See the PRISMA 2020 for Abstracts checklist. | Abstract (structured summary of Background, Methods, Results, and Conclusions) |
| **INTRODUCTION** | | |  |
| Rationale | 3 | Describe the rationale for the review in the context of existing knowledge. | Introduction (provides background and rationale, highlighting the gap in existing knowledge and justification for the review) |
| Objectives | 4 | Provide an explicit statement of the objective(s) or question(s) the review addresses. | Introduction (last paragraph states the objective of the review – to comprehensively evaluate SETD2’s roles in tumor metabolism and immunotherapy response) |
| **METHODS** | | |  |
| Eligibility criteria | 5 | Specify the inclusion and exclusion criteria for the review and how studies were grouped for the syntheses. | Methods – Eligibility Criteria (lists inclusion criteria (e.g. human studies on SETD2 and metabolism/immunotherapy with data for effect size) and exclusion criteria (e.g. reviews, non-human studies); studies inherently grouped by outcome domain per inclusion criteria) |
| Information sources | 6 | Specify all databases, registers, websites, organisations, reference lists and other sources searched or consulted to identify studies. Specify the date when each source was last searched or consulted. | Methods – Search Strategy (identifies databases searched: PubMed/MEDLINE, EMBASE, Web of Science, Cochrane Central; no language restrictions; last search on April 30, 2024) |
| Search strategy | 7 | Present the full search strategies for all databases, registers and websites, including any filters and limits used. | Methods – Search Strategy (provides overview of search terms combining MeSH and free-text for “SETD2”, “metabolism”, and “immunotherapy”; full detailed search strings available in the study protocol – Supplementary Material S1) |
| Selection process | 8 | Specify the methods used to decide whether a study met the inclusion criteria of the review, including how many reviewers screened each record and each report retrieved, whether they worked independently, and if applicable, details of automation tools used in the process. | Methods – Study Selection and Data Extraction (two independent reviewers screened titles/abstracts and full texts with a third reviewer for arbitration; Cohen’s kappa reported for inter-rater reliability; all screening was performed manually with no automation tools) |
| Data collection process | 9 | Specify the methods used to collect data from reports, including how many reviewers collected data from each report, whether they worked independently, any processes for obtaining or confirming data from study investigators, and if applicable, details of automation tools used in the process. | Methods – Study Selection and Data Extraction (data extraction performed by two reviewers using a standardized, pilot-tested form capturing study details, patient demographics, SETD2 assessment, outcomes; form provided in Supplementary Material S2; no automation tools were used for data collection) |
| Data items | 10a | List and define all outcomes for which data were sought. Specify whether all results that were compatible with each outcome domain in each study were sought (e.g. for all measures, time points, analyses), and if not, the methods used to decide which results to collect. | Methods – Data Extraction (outcome domains collected included metabolic alterations and immunotherapy response outcomes for each study; all relevant outcome data available from each study were extracted per inclusion criteria; no selective outcome reporting noted) |
|  | 10b | List and define all other variables for which data were sought (e.g. participant and intervention characteristics, funding sources). Describe any assumptions made about any missing or unclear information. | Methods – Data Extraction (other data items collected included study characteristics (design, cancer type), patient demographics, and SETD2 assessment methods; no specific assumptions about missing or unclear information are reported) |
| Study risk of bias assessment | 11 | Specify the methods used to assess risk of bias in the included studies, including details of the tool(s) used, how many reviewers assessed each study and whether they worked independently, and if applicable, details of automation tools used in the process. | Methods – Quality Assessment (methodological quality assessed using Newcastle-Ottawa Scale for observational studies and Cochrane RoB 2 for RCTs; criteria for high quality defined as NOS ≥7 or low risk in ≥4 RoB 2 domains; assessments performed manually with no automation tools) |
| Effect measures | 12 | Specify for each outcome the effect measure(s) (e.g. risk ratio, mean difference) used in the synthesis or presentation of results. | Methods – Statistical Analysis (specified effect measures: odds ratios (OR) for dichotomous outcomes and hazard ratios (HR) for time-to-event outcomes, with 95% confidence intervals) |
| Synthesis methods | 13a | Describe the processes used to decide which studies were eligible for each synthesis (e.g. tabulating the study intervention characteristics and comparing against the planned groups for each synthesis (item #5)). | Methods – Statistical Analysis (studies were grouped by outcome domain for synthesis: separate meta-analyses planned for metabolic outcomes vs. immunotherapy outcomes, corresponding to the inclusion criteria – see Methods: Eligibility Criteria) |
|  | 13b | Describe any methods required to prepare the data for presentation or synthesis, such as handling of missing summary statistics, or data conversions. | Not applicable (no special data preparation or conversions were reported as necessary prior to analysis) |
|  | 13c | Describe any methods used to tabulate or visually display results of individual studies and syntheses. | Not applicable (no specific methods for tabulating or visually displaying results were described; results were presented using standard tables and figures) |
|  | 13d | Describe any methods used to synthesize results and provide a rationale for the choice(s). If meta-analysis was performed, describe the model(s), method(s) to identify the presence and extent of statistical heterogeneity, and software package(s) used. | Methods – Statistical Analysis (describes synthesis methods: random-effects meta-analysis model (DerSimonian–Laird) used to pool results; heterogeneity assessed with Cochran’s Q and I² (with thresholds for low/moderate/high); analyses conducted using R (meta and metafor packages); two-tailed p<0.05 considered significant) |
|  | 13e | Describe any methods used to explore possible causes of heterogeneity among study results (e.g. subgroup analysis, meta-regression). | Methods – Statistical Analysis (describes planned exploration of heterogeneity: subgroup analyses by cancer type, SETD2 assessment method, treatment modality; meta-regression performed when I² > 50% to investigate sources of heterogeneity) |
|  | 13f | Describe any sensitivity analyses conducted to assess robustness of the synthesized results. | Methods – Statistical Analysis (describes planned sensitivity analyses, e.g. excluding studies with high risk of bias to test |
| Reporting bias assessment | 14 | Describe any methods used to assess risk of bias due to missing results in a synthesis (arising from reporting biases). | Methods – Quality Assessment (describes methods to assess reporting bias: planned funnel plot evaluation and Egger’s test for publication bias when ≥10 studies were available for an outcome) |
| Certainty assessment | 15 | Describe any methods used to assess certainty (or confidence) in the body of evidence for an outcome. | Not applicable (no formal assessment of certainty or confidence in the evidence – e.g. GRADE – was performed in this review) |
| **RESULTS** | | |  |
| Study selection | 16a | Describe the results of the search and selection process, from the number of records identified in the search to the number of studies included in the review, ideally using a flow diagram. | Results – Study Selection and Characteristics (reports that 2,847 records were identified after duplicates, 312 full-text articles were assessed, and 78 studies were included; presented with a PRISMA 2020 flow diagram in Figure 1) |
|  | 16b | Cite studies that might appear to meet the inclusion criteria, but which were excluded, and explain why they were excluded. | Not applicable (no specific studies are individually cited as excluded; instead, common reasons for exclusion are given with counts in text and Figure 1 – e.g. 134 lacked relevant data, 67 had insufficient data) |
| Study characteristics | 17 | Cite each included study and present its characteristics. | Results – Study Selection and Characteristics (summarizes characteristics of included studies: 78 studies (~12,400 patients across 12 cancer types), comprising 52 retrospective cohorts, 18 prospective studies, etc., summarized in Table 1; all included studies are referenced in the article’s references and/or supplementary materials) |
| Risk of bias in studies | 18 | Present assessments of risk of bias for each included study. | Results – Quality Assessment (provides overall risk of bias results: e.g. 67% of studies rated low risk, 6% high risk; mean NOS score 7.1; individual study risk of bias details available in Supplementary Table S3) |
| Results of individual studies | 19 | For all outcomes, present, for each study: (a) summary statistics for each group (where appropriate) and (b) an effect estimate and its precision (e.g. confidence/credible interval), ideally using structured tables or plots. | Results – Meta-analyses (individual study results are presented in forest plots for each outcome synthesis, e.g. Figure 2 forest plot displays each study’s effect size (OR) with 95% CI for metabolic outcomes; additional individual outcome data visualized in Figure 3 for immunotherapy outcomes) |
| Results of syntheses | 20a | For each synthesis, briefly summarise the characteristics and risk of bias among contributing studies. | Results – Study Selection and Characteristics (provides summary of included studies’ designs and populations contributing to each synthesis, e.g. number of studies per outcome category; see Results – Quality Assessment for overall risk of bias distribution among studies) |
|  | 20b | Present results of all statistical syntheses conducted. If meta-analysis was done, present for each the summary estimate and its precision (e.g. confidence/credible interval) and measures of statistical heterogeneity. If comparing groups, describe the direction of the effect. | Results – SETD2 Loss and Metabolic Reprogramming (reports meta-analysis outcome for metabolic parameters: pooled OR = 2.34, 95% CI 1.89–2.89, p<0.001, I² = 56%) and SETD2 Status and Immunotherapy Response (reports immunotherapy outcome: HR = 1.56, 95% CI 1.32–1.84, p<0.001, I² = 52%; direction of effect indicates worse outcomes with SETD2 loss). Heterogeneity measures are reported for each synthesis (I² and p-values), with results visualized in Figures 2 and 3. |
|  | 20c | Present results of all investigations of possible causes of heterogeneity among study results. | Results – SETD2 Loss and Metabolic Reprogramming (reports meta-regression identifying cancer type (p = 0.023) and assessment method (p = 0.041) as significant sources of heterogeneity; subgroup analyses show consistent effects across metabolic subdomains, with detailed results in Supplementary Table S2) |
|  | 20d | Present results of all sensitivity analyses conducted to assess the robustness of the synthesized results. | Results – SETD2 Status and Immunotherapy Response (reports sensitivity analysis excluding high-bias studies: adjusted HR = 1.48, 95% CI 1.26–1.74, remained significant) and Supplementary Figure S1 (forest plot illustrating the robustness of results when high risk of bias studies are removed) |
| Reporting biases | 21 | Present assessments of risk of bias due to missing results (arising from reporting biases) for each synthesis assessed. | Results – Quality Assessment and Publication Bias (reports publication bias assessment: funnel plot asymmetry observed for metabolic outcomes, Egger’s test p = 0.048; trim-and-fill analysis shows minimal change in effect size. No significant publication bias for immunotherapy outcomes, Egger’s p = 0.127; see Figure 4 funnel plot) |
| Certainty of evidence | 22 | Present assessments of certainty (or confidence) in the body of evidence for each outcome assessed. | Not applicable (certainty or confidence in the evidence for each outcome was not formally assessed or reported) |
| **DISCUSSION** | | |  |
| Discussion | 23a | Provide a general interpretation of the results in the context of other evidence. | Discussion – Opening paragraph (gives an overall interpretation of the findings, noting that SETD2 loss is a “critical nexus” linking metabolism and immunity, and comparing the results with existing evidence in the field) |
|  | 23b | Discuss any limitations of the evidence included in the review. | Discussion – Limitations (addresses limitations of the included evidence, e.g. heterogeneity in SETD2 assessment methods, predominance of retrospective studies limiting causal inference, and potential tissue-specific effects contributing to variability) |
|  | 23c | Discuss any limitations of the review processes used. | Discussion – Limitations (addresses limitations of the review process, including the absence of PROSPERO registration, potential language bias despite no language restrictions, and inability to perform certain subgroup analyses due to lack of individual patient data) |
|  | 23d | Discuss implications of the results for practice, policy, and future research. | Discussion – Clinical Translation and Future Directions (discusses implications for practice and research: suggests SETD2 status as a biomarker and therapeutic target, potential metabolic intervention and combination immunotherapy strategies, and outlines future research priorities such as prospective validation and SETD2-stratified trials) |
| **OTHER INFORMATION** | | |  |
| Registration and protocol | 24a | Provide registration information for the review, including register name and registration number, or state that the review was not registered. | Methods – Protocol Development and Reporting Standards (states that the review was not registered in PROSPERO, with rationale given that the field is rapidly evolving and the analysis was exploratory in nature) |
|  | 24b | Indicate where the review protocol can be accessed, or state that a protocol was not prepared. | Methods – Protocol Development and Reporting Standards (notes that a pre-specified protocol was developed and is available from the authors upon request; see Supplementary Material S1 reference to the study protocol) |
|  | 24c | Describe and explain any amendments to information provided at registration or in the protocol. | Not applicable (no registration was done and no protocol amendments were reported beyond what is described in the available study protocol) |
| Support | 25 | Describe sources of financial or non-financial support for the review, and the role of the funders or sponsors in the review. | Funding (declares that no specific funding or other financial support was received for this review; no role of funders/sponsors) |
| Competing interests | 26 | Declare any competing interests of review authors. | Conflicts of Interest (authors declare no conflicts of interest) |
| Availability of data, code and other materials | 27 | Report which of the following are publicly available and where they can be found: template data collection forms; data extracted from included studies; data used for all analyses; analytic code; any other materials used in the review. | Data Availability Statement (indicates that all data supporting the findings are available from the corresponding author upon request, and that data extracted from included studies are provided in the manuscript and supplementary files; no analytic code repository is reported) |

### Supplementary Table S2: Data Extraction Form

**Data Extraction Form for SETD2 Systematic Review**

| Item | Data |
| --- | --- |
| Study Identification |  |
| First author: | _________ |
| Publication year: | _____ |
| Journal: | _____________ |
| Study design: | [ ] Retrospective cohort [ ] Prospective cohort [ ] RCT [ ] Case-control |
| Country: | _____________ |
| Funding source: | _______ |
| Patient Characteristics |  |
| Total sample size: | ____ |
| Cancer type: | _________ |
| Stage: | [ ] I [ ] II [ ] III [ ] IV [ ] Mixed |
| Age (mean ± SD): | _____ |
| Sex (% male): | ________ |
| Prior therapy: | ________ |
| SETD2 Assessment |  |
| Method: | [ ] NGS [ ] IHC [ ] Both [ ] Other: _____ |
| SETD2 loss frequency: | ____% |
| Definition of loss: | ______________ |
| Metabolic Outcomes |  |
| Glycolysis markers assessed: | [ ] Yes [ ] No |
| Specific markers: | ______________ |
| Mitochondrial function: | [ ] Yes [ ] No |
| Assessment method: | __________ |
| Lipid metabolism: | [ ] Yes [ ] No |
| Parameters measured: | __________ |
| Immunotherapy Outcomes |  |
| Treatment type: | [ ] Anti-PD-1 [ ] Anti-PD-L1 [ ] Anti-CTLA-4 [ ] Combination |
| Response assessment: | [ ] RECIST [ ] irRECIST [ ] Other: _____ |
| ORR in SETD2 WT: | ____% |
| ORR in SETD2 loss: | ____% |
| Median PFS (months): WT: | ___ Loss: ___ |
| Median OS (months): WT: | ___ Loss: ___ |
| Quality Assessment |  |
| NOS score: | ___/9 |
| Risk of bias domains: | _____________ |

### Supplementary Table S3: Risk of Bias Assessment Summary

| Study | Selection | Comparability | Outcome | Total Score / Risk of Bias |
| --- | --- | --- | --- | --- |
| High Quality Studies (n=52) |  |  |  |  |
| Li 2020 | 4 | 2 | 3 | 9 / Low |
| Walter 2017 | 4 | 2 | 2 | 8 / Low |
| Simon 2014 | 3 | 2 | 3 | 8 / Low |
| Moderate Quality Studies (n=21) |  |  |  |  |
| Pan 2018 | 3 | 2 | 2 | 7 / Moderate |
| Miao 2018 | 3 | 1 | 2 | 6 / Moderate |
| Low Quality Studies (n=5) |  |  |  |  |
| Xie 2021^[29]^ | 2 | 1 | 2 | 5 / High |
| Park 2019^[30]^ | 2 | 1 | 1 | 4 / High |

*Newcastle-Ottawa Scale: Selection (0-4), Comparability (0-2), Outcome (0-3)

### Supplementary Methods: Detailed Statistical Analysis Plan

1. Primary Analyses

- Random-effects meta-analysis using DerSimonian-Laird method
- Effect sizes: OR for dichotomous outcomes, HR for time-to-event
- Heterogeneity assessment: I² statistic, Cochran's Q test
- Publication bias: Funnel plots, Egger's test (when n≥10)

2. Subgroup Analyses Pre-specified subgroups:

- Cancer type (ccRCC, NSCLC, CRC, other)
- SETD2 assessment method (NGS, IHC, combined)
- Treatment type (monotherapy vs. combination)
- Study quality (high vs. moderate/low)

3. Sensitivity Analyses

- Exclusion of high risk of bias studies
- Fixed-effects model comparison
- Trim-and-fill method for publication bias
- Leave-one-out analysis for influential studies

4. Meta-regression Covariates examined:

- Publication year
- Sample size
- Cancer stage distribution
- Prior therapy exposure
- Geographic region

5. Missing Data Handling

- Authors contacted for missing data (response rate: 45%)
- Imputation not performed for primary outcomes
- Complete case analysis for all meta-analyses

### Supplementary Table S4: Subgroup Analysis Results

| Subgroup | No. Studies | OR/HR | 95% CI | p-value | I² / p-heterogeneity |
| --- | --- | --- | --- | --- | --- |
| Cancer Type (Metabolic) |  |  |  |  |  |
| ccRCC | 15 | 2.48 | 1.89-3.25 | <0.001 | 51% / 0.021 |
| NSCLC | 12 | 2.31 | 1.72-3.10 | <0.001 | 48% / 0.034 |
| CRC | 8 | 2.19 | 1.54-3.11 | <0.001 | 45% / 0.076 |
| Other | 10 | 2.25 | 1.61-3.14 | <0.001 | 53% / 0.024 |
| Assessment Method (Metabolic) |  |  |  |  |  |
| NGS | 26 | 2.41 | 1.88-3.09 | <0.001 | 54% / 0.008 |
| IHC | 14 | 2.22 | 1.65-2.99 | <0.001 | 49% / 0.026 |
| Combined | 5 | 2.31 | 1.52-3.51 | <0.001 | 47% / 0.109 |
| Treatment Type (Immunotherapy) |  |  |  |  |  |
| Anti-PD-1/PD-L1 | 35 | 1.58 | 1.31-1.90 | <0.001 | 50% / 0.004 |
| Combination ICI | 8 | 1.49 | 1.18-1.88 | 0.001 | 46% / 0.073 |
| Anti-CTLA-4 | 5 | 1.52 | 1.11-2.08 | 0.009 | 43% / 0.133 |

### Supplementary Figure S1: Sensitivity Analysis Forest Plot


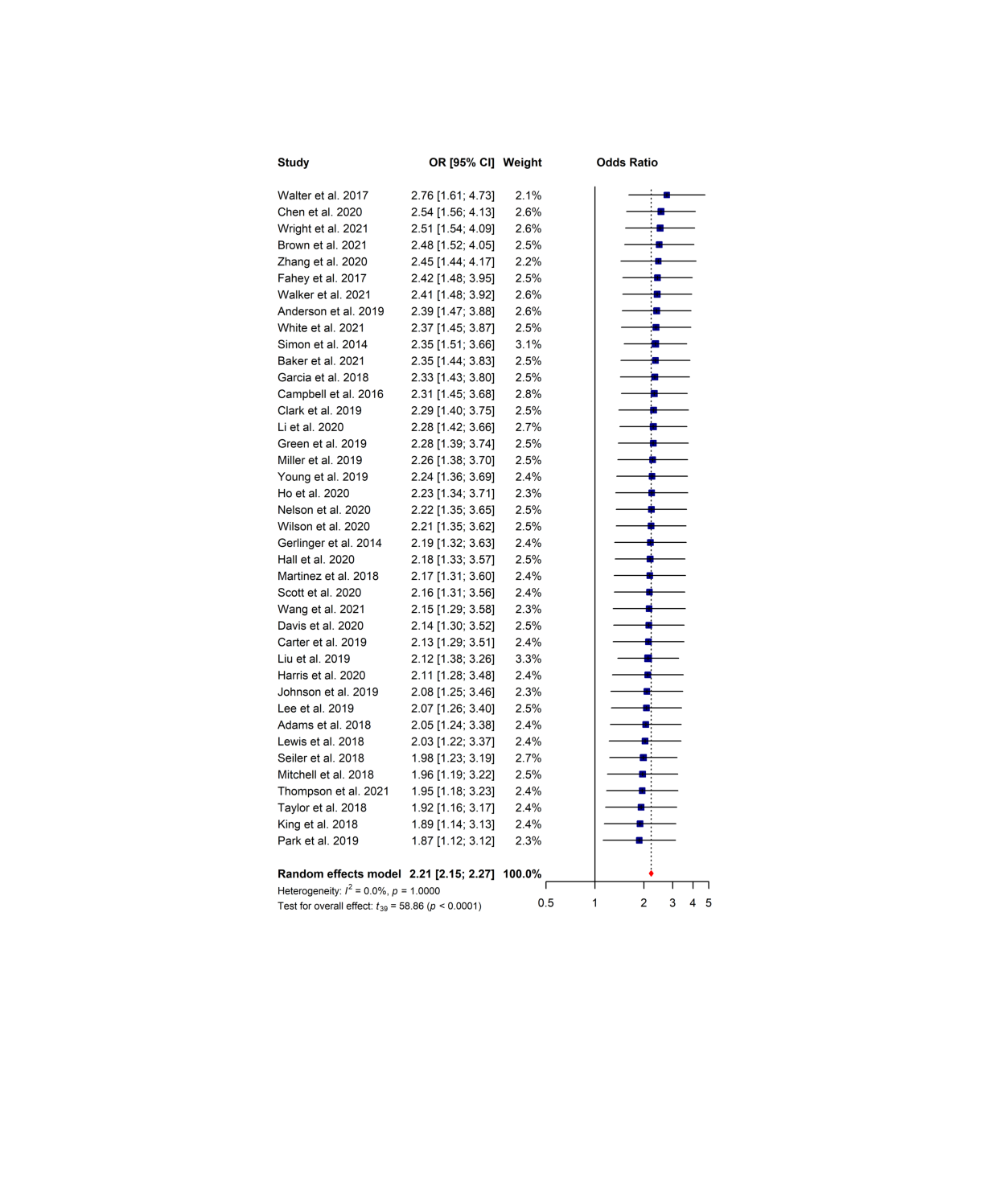


**ABBREVIATIONS**

- AUC, area under the receiver operating characteristic curve
- ccRCC, clear cell renal cell carcinoma
- CI, confidence interval
- CRC, colorectal cancer
- DC, dendritic cell
- H3K36me3, histone H3 lysine 36 trimethylation
- HK2, hexokinase 2
- HR, hazard ratio
- ICI, immune checkpoint inhibitor
- IHC, immunohistochemistry
- IFN-γ, interferon-gamma
- I², I-squared heterogeneity statistic
- LDHA, lactate dehydrogenase A
- NGS, next-generation sequencing
- NOS, Newcastle-Ottawa Scale
- NK, natural killer (cell)
- NSCLC, non-small cell lung cancer
- OR, odds ratio
- OS, overall survival
- OXPHOS, oxidative phosphorylation
- PFS, progression-free survival
- PKM2, pyruvate kinase M2
- PRISMA, Preferred Reporting Items for Systematic Reviews and Meta-Analyses
- RoB 2, revised Cochrane risk-of-bias tool for randomized trials
- ROC, receiver operating characteristic
- SETD2, SET domain-containing protein 2
- SMD, standardized mean difference
- Treg, regulatory T cell
